# Venous Thromboembolism Risk Assessment and Prophylaxis Administration among Admitted Patients in Lagos, Nigeria: A Quality Improvement Project

**DOI:** 10.4314/ejhs.v35i3.5

**Published:** 2025-05

**Authors:** Ugochi Chinenye Okorafor, Uchechi Chioma Okorafor

**Affiliations:** 1 Department of Cardiology, Meridian Cardiac Center, Lagos, Nigeria. Postal Address: Meridian Cardiac Center, 5th Avenue, A Close, House 8, Festac Town, Lagos, Nigeria, 102312; 2 Department of Medicine and Surgery, College of Medicine, University of Ibadan, Ibadan, Nigeria. Postal Address: College of Medicine, University of Ibadan, Ibadan, Nigeria, 200221

**Keywords:** Clinical audit, Prophylaxis, Quality Improvement, Risk assessment, Venous Thromboembolism

## Abstract

**Background:**

Venous thromboembolism (VTE) is a potentially life-threatening and costly complication of hospitalization. In many developing countries, VTE risk assessment at admission is not routinely conducted. This quality improvement project aimed to audit and improve the VTE risk assessment and prophylaxis administration among patients admitted to the Meridian Cardiac Centre in Lagos, Nigeria, over two cycles.

**Methods:**

This was a quality improvement project. The National Institute for Health and Clinical Excellence (NICE) guideline NG89 served as the standard. In the second cycle, the Caprini Risk Assessment Model (RAM) was introduced for assessing VTE risk at admission. The RAM was also retrospectively applied to assess patients' VTE risk categories in both cycles.

**Results:**

No patients were assessed for their VTE risk during the first cycle. By the second cycle, following an educational meeting and the introduction of the Caprini RAM, 56% of patients were assessed for their VTE risk.

**Conclusions:**

VTE risk assessment practices were poor in this facility. Continuous education for medical practitioners and proper documentation of clinical notes will improve the categorization of medical inpatients based on their VTE risk and improve the outcomes of future audits.

## Introduction

Venous thromboembolism (VTE) is a known risk associated with immobilization in hospitalized patients. VTE, which encompasses deep venous thrombosis (DVT) and pulmonary embolism (PE), is the third leading cause of death from a cardiovascular condition (1). Approximately 1 in 5 affected patients die within a year of diagnosis (2). Pulmonary embolism is the most common preventable cause of hospital deaths worldwide (3). In addition to its health implications, VTE is an expensive disease, with the estimated annual cost of VTE-related care in the United States ranging from $2 billion to $10 billion (4). VTE affects individuals of all ages, but it is particularly prevalent among the elderly (1), a group at high risk for hospitalization due to comorbidities. Data on its actual prevalence in Sub-Saharan Africa is limited, although the incidence is believed to be higher among Blacks (5).

Despite the availability of statistics on the implications of VTE and the demonstrated impact of routine risk assessment models (RAMs) in preventing VTE-related morbidity and mortality, mechanical and pharmacologic thromboprophylaxis prescriptions are still not routine in many parts of the world. Globally, about half of all hospitalized patients are at risk of VTE, but only 54.5% of them receive adequate thromboprophylaxis (6). In Africa, this figure is even lower, with only 44.9% of at-risk patients receiving appropriate prophylaxis (6). Data on VTE risk stratification practices among Nigerian patients is sparse, highlighting the need for more research, particularly among medical inpatients. Data from Enugu, Nigeria, suggests that VTE risk is high among both surgical and medical inpatients, with over 50% of admitted patients being at risk (7).

Current evidence shows that RAMs are generally weak in predicting VTE, with no single model proving superior to others (8). However, data supporting the routine use of VTE risk assessment to prevent hospital readmissions and deaths is available. The United Kingdom's National Institute for Health and Care Excellence (NICE) recommends formal VTE risk assessments for all hospitalized patients, with the prescription of appropriate prophylaxis (9). In 2010, the National Health Service (NHS) set a national target of 90% compliance for VTE risk assessment using a standardized tool, which led to a decrease in VTE-related mortalities and hospital readmission rates (10-12). The International Society on Thrombosis and Haemostasis (ISTH) has called for the incorporation of VTE risk assessments into hospital and national policies, with outcomes research to demonstrate their effectiveness (10). This project was conducted in response to the ISTH's call, with the goal of improving local practices through the implementation of the Caprini RAM. This work was previously presented as a poster at the British Cardiovascular Society Conference in Manchester, United Kingdom, from June 3-5, 2024 (13).

## Materials and Methods

This study was conducted at a privately owned cardiology hospital in Lagos, Nigeria. It was designed and reported as a quality improvement project (QIP) with two cycles, following SQUIRE 2.0 guidelines (14). Permission was obtained from hospital authorities to access patient data. Since the data was collected from clinical notes and no direct patient contact was required, human research ethics approval was not sought. The study adhered to the principles of the Helsinki Declaration.

In the first cycle, all patients aged 18 years and older admitted in September and October 2023 had their case notes reviewed for sociodemographic data and VTE risk factors. The case data collection form was designed according to the National Institute for Health and Clinical Excellence (NICE) standard NG89 (15). Patient admission data was obtained from the nurses' admission register. Table 1 outlines the information collected from each case note. De-identified data was entered into a Microsoft Excel spreadsheet and analyzed using the Statistical Package for the Social Sciences (SPSS) version 21 to assess the hospital's performance against the specified criteria. The target was set for 80% compliance, but no statistical tests were conducted.

**Table 1:** Data collection form

➢	Case ID number
➢	Age
➢	Sex
➢	Diagnosis
➢	Number of days spent on admission
➢	Did the patient have assessment of VTE risk as soon as possible after admission? Yes or No
➢	Did the patient receive VTE prophylaxis? Yes or No
➢	Number of days VTE prophylaxis was administered for
➢	Date and Time of Admission
➢	Was the first dose of thromboprophylaxis within 14 hours of admission? Yes or No

The Caprini RAM, published in 2005 (16,17), was introduced after a meeting with doctors at the facility, where the findings from the first audit were shared. The RAM was chosen due to its availability at the hospital, which minimized costs, and was placed at doctors' consulting desks for easy access during documentation. Implementation of the RAM began on December 1, 2023, and continued for 10 weeks until February 9, 2024. The model was to be used for all admitted patients aged 18 and above, either at admission or at the first consultant review. Based on the VTE risk score, appropriate prophylaxis was to be administered, with exceptions noted in the case notes.

A second retrospective analysis, using the same data collection form, was conducted to assess improvements in VTE risk assessment and prophylaxis administration. The RAM was also used to retrospectively assess the VTE risk categories of patients who had not been assessed at admission. The cutoff scores for the Caprini RAM were: low risk (0 or 1), moderate risk (2), high risk (3 or 4), and highest risk (5 or more) (16).

## Results

The two cycles involved a total of 44 patients, with 26 patients in the first cycle and 18 patients in the second cycle. The mean age of patients in the first cycle was 56.58±13.76 years, while in the second cycle, it was 57.44±16.02 years. In the first cycle, no patients (0%) had their VTE risk assessed at any point during admission. Retrospective assessment using the Caprini RAM showed that 88% of patients were at risk of VTE (moderate, high, or highest risk) and were eligible for prophylaxis. Of these, 57% received thromboprophylaxis during their hospital stay. In the second cycle, 56% of patients had their VTE risk assessed at admission, of which at-risk patients comprised 94% of the total number of patients assessed. 65% of at-risk patients received prophylaxis. Table 2 provides the detailed results of the QIP.

**Table 2:** Description of results by cycle

VARIABLES	CYCLE 1 (N=26)	CYCLE 2 (N=18)
	N (%)	N (%)
Gender		
Male	7(27)	11(61)
Female	19(73)	7(39)
Diagnosis/Admission Cause		
Heart Failure	9(35)	12(67)
Others (e.g. acute infective exacerbation of asthma/COPD, stroke, sepsis, symptomatic atrial flutter, pulmonary embolism, acute gastritis)	17(65)	6(33)
Was VTE Risk Assessment Completed?		
Yes	0(0)	10(56)
No	26(100)	8(44)
Risk Level		
Highest	11(42)	8(44)
High	7(27)	5(28)
Moderate	5(19)	4(22)
Low	3(12)	1(6)
At-Risk Patients Who Received Prophylaxis?	**N=23**	**N=17**
Yes	13(57)	11(65)
No	9(39)	2(12)
Na (Already on Rivaroxaban)	1(4)	4(23)
First Dose of Prophylaxis Given Within 14 Hours?	**N=13**	**N=11**
Yes	8(61)	8(73)
No	1(8)	-
NI	4(31)	3(27)
Number Of Days Spent on Admission	4.31±2.71	5.11±2.30

NA: Not applicable; NI: No information; VTE: Venous thromboembolism.

## Discussion

VTE is a common complication of hospitalization, but data on the current state of VTE risk assessment in Nigerian medical inpatients is limited. This project aimed to address this gap and demonstrate the impact of a simple intervention—educating healthcare personnel—on improving VTE risk assessment and prophylaxis administration.

In the first cycle, no patients had their VTE risk assessed. When retrospective Caprini scores were calculated, 88% were found to be at risk of VTE (Caprini ≥2). This figure is higher than some studies but lower than others, such as Nkoke et al. (18), who observed a 92.8% rate of VTE risk, and Beutel et al. (3), who reported a 34.4% rate. Regarding prophylaxis administration, 57% of at-risk patients received thromboprophylaxis, although no prescriptions for mechanical prophylaxis were made. This rate is higher than some studies, but lower than Beutel et al.'s report of 4.6% (3).

In the second cycle, 94% of patients were at risk for VTE, with 65% receiving prophylaxis. This increase in at-risk patients could be attributed to the nature of the admissions, with heart failure being the most common diagnosis (67%). However, no prescriptions for mechanical prophylaxis were made.

The project highlighted the lack of structured VTE risk assessment in this facility prior to the intervention. Other Nigerian studies have shown that many physicians do not carry out risk stratification or follow guidelines for prophylaxis administration (22). A global survey by the ISTH in 2019 revealed that only 8.8% of the 34 countries surveyed had national guidelines recommending routine VTE risk assessment (23). The findings of this study underscore the need for improving VTE risk assessment practices and the routine use of RAMs in hospitals, particularly in developing countries like Nigeria.

In conclusion, this quality improvement project demonstrated the poor state of VTE risk assessment in the facility, with no patients assessed in the first cycle. The introduction of the Caprini RAM, coupled with an educational meeting for healthcare personnel, increased the number of VTE risk assessments and the number of at-risk patients receiving prophylaxis. While the results did not meet the desired targets, the intervention showed promise, and its low cost makes it a feasible option for broader implementation in Nigeria. It is hoped that this project encourages the routine use of VTE risk assessment in hospitals nationwide.

A limitation of this study is the small sample size, which limits the generalizability of the results. Additionally, incomplete documentation of vital details, such as age, sex, and the time of admission and prophylaxis administration, made it difficult to assess some aspects of the intervention's impact. The use of electronic medical records, which is currently being introduced at the facility, is expected to address these documentation issues. Furthermore, the presence of doctors working locum shifts during the intervention period may have hindered the widespread adoption of the Caprini RAM. Future audits should aim to address these limitations and continue improving VTE risk assessment practices.
